# Pain and mild cognitive impairment among adults aged 50 years and above residing in low- and middle-income countries

**DOI:** 10.1007/s40520-023-02434-7

**Published:** 2023-05-25

**Authors:** Lee Smith, Guillermo F. López Sánchez, Jae Il Shin, Pinar Soysal, Damiano Pizzol, Yvonne Barnett, Karel Kostev, Louis Jacob, Nicola Veronese, Laurie Butler, Helen Odell-Miller, Jodie Bloska, Benjamin R. Underwood, Ai Koyanagi

**Affiliations:** 1 Centre for Health Performance and Wellbeing, Anglia Ruskin University, Cambridge, UK; 2 Division of Preventive Medicine and Public Health, Department of Public Health Sciences, School of Medicine, University of Murcia, Murcia, Spain; 3 Department of Pediatrics, Yonsei University College of Medicine, Seoul, South Korea; 4 Department of Geriatric Medicine, Faculty of Medicine, Bezmialem Vakif University, Istanbul, Turkey; 5 Italian Agency for Development Cooperation, Khartoum, Sudan; 6 University Clinic of Marburg, Marburg, Germany; 7 Research and Development Unit, Parc Sanitari Sant Joan de Déu, CIBERSAM, ISCIII, Dr. Antoni Pujadas, Sant Boi de Llobregat, Barcelona, Spain; 8 Department of Physical and Rehabilitation Medicine, Lariboisière-Fernand Widal Hospital, AP-HP, University Paris Cité, Paris, France; 9 Department of Internal Medicine, Geriatrics Section, University of Palermo, Palermo, Italy; 10 Cambridge Institute for Music Therapy Research, Anglia Ruskin University, Cambridge, UK; 11 Department of Psychiatry, University of Cambridge, Herchel Smith Building, Forvie Site, Robinson Way, Cambridge , CB2 0SZ UK; 12 Cambridgeshire and Peterborough NHS Foundation Trust, Windsor Unit, Fulbourn Hospital, Cambridge, CB21 5EF UK; 13 ICREA, Pg. Lluis Companys 23, 08010 Barcelona, Spain

**Keywords:** Pain, Mild cognitive impairment, Low- and middle-income countries, Epidemiology

## Abstract

**Background:**

Previous studies on the association between pain and cognitive decline or impairment have yielded mixed results, while studies from low- and middle-income countries (LMICs) or specifically on mild cognitive impairment (MCI) are scarce. Thus, we investigated the association between pain and MCI in LMICs and quantified the extent to which perceived stress, sleep/energy problems, and mobility limitations explain the pain/MCI relationship.

**Methods:**

Data analysis of cross-sectional data from six LMICs from the Study on Global Ageing and Adult Health (SAGE) were performed. MCI was based on the National Institute on Aging-Alzheimer's Association criteria. "Overall in the last 30 days, how much of bodily aches or pain did you have?” was the question utilized to assess pain. Associations were examined by multivariable logistic regression analysis and meta-analysis.

**Results:**

Data on 32,715 individuals aged 50 years and over were analysed [mean (SD) age 62.1 (15.6) years; 51.7% females]. In the overall sample, compared to no pain, mild, moderate, and severe/extreme pain were dose-dependently associated with 1.36 (95% CI = 1.18–1.55), 2.15 (95% CI = 1.77–2.62), and 3.01 (95% CI = 2.36–3.85) times higher odds for MCI, respectively. Mediation analysis showed that perceived stress, sleep/energy problems, and mobility limitations explained 10.4%, 30.6%, and 51.5% of the association between severe/extreme pain and MCI.

**Conclusions:**

Among middle-aged to older adults from six LMICs, pain was associated with MCI dose-dependently, and sleep problems and mobility limitations were identified as potential mediators. These findings raise the possibility of pain as a modifiable risk factor for developing MCI.

## Introduction

Dementia is a syndrome characterized by deterioration in cognitive function beyond what might be expected from the usual consequences of biological ageing, which impacts on function [1]. Globally, approximately 55 million people are living with dementia, with over 60% of these people living in low- and middle-income countries (LMICs). Owing to the proportion of older adults increasing in nearly every country, the number of dementia cases is expected to increase to approximately 78 million in 2030, and 139 million in 2050 [1]. Such a high and rising rate of dementia is a significant problem as it is one of the major causes of morbidity and mortality in older adults, and its social and economic impacts are substantial. For example, in 2019, the estimated total global societal cost of dementia was US$ 1.3 trillion, and these costs are expected to surpass US$ 2.8 trillion by 2030 as both the number of people living with dementia and care costs increase [1].

However, to date, there is no treatment for any form of dementia which slows disease progression. Therefore, it is of utmost importance to identify risk factors that can be modified at the precursory stage of dementia to inform targeted intervention to prevent or delay the onset of dementia. Mild cognitive impairment (MCI) is a preclinical state of dementia with annual conversion rates to dementia ranging from 10 to 15% in clinical samples and 3.8% to 6.3% in community-based samples [2]. MCI is considered an important target for intervention in the prevention or delay of dementia.

One understudied but potentially important risk factor for MCI in the context of LMICs is pain. The prevalence of pain increases with age and can be particularly high in LMICs due to limited availability of proper treatment. For example, the World Health Organization (WHO) estimates that 5.5 billion people (more than 80% of the global population) do not have access to treatments for moderate to severe pain and that most of these people live in LMICs [3]. Pain may plausibly increase risk for MCI via several mechanisms. For instance, perceived stress, sleep problems, and low physical activity due to mobility limitations can all be consequences of pain, and all these factors have been reported to be risk factors for MCI [4–11]. Furthermore, pain has been found to be associated with brain plasticity and structural changes in different cortical regions associated with learning, memory, fear, and emotional responses [12]. However, previous studies on the association between pain and cognitive decline or impairment have yielded conflicting results, with some studies finding positive associations while others no associations, and this may be due to the different definitions of cognitive decline employed [13, 14]. Importantly, to date, the relationship between pain and MCI that is distinctly different from general cognitive decline (which may not necessarily be related with higher risk for future dementia onset) has not been examined. Clearly, investigations from diverse settings are required. Moreover, it is important to elucidate the potential mediators in the association between pain and MCI for targeted prevention efforts. Given this background, the aim of the present study was to investigate the association between pain and MCI in a representative sample of 32,715 individuals aged 50 years and over from six LMICs. A further aim was to identify to what extent perceived stress, sleep/energy problems, and mobility limitations may explain the association between pain and MCI.

## Methods

Secondary data analysis of the Study on Global Ageing and Adult Health (SAGE) 2007–2010 was performed. The main aim of this survey was to obtain comparable and valid information on wellbeing and health among middle-aged and older adults. China, Ghana, India, Mexico, Russia, and South Africa were the countries that participated in the survey. Of note, two of the most populous countries in the world were included (i.e., China and India). Based on the World Bank classification when the survey was conducted, Ghana and India were a low-income country and a lower middle-income country, respectively, while Mexico, Russia, and South Africa were upper middle-income countries. China was a lower middle-income country at the beginning of the survey but became an upper middle-income country in 2010. Details of the survey methodology have been published elsewhere [15]. In brief, a multistage clustered sampling design method was employed to obtain samples which are nationally representative. The sample consisted of adults aged 18 years and over, while people aged 50 years and over were oversampled. Trained interviewers performed face-to-face interviews utilizing a standard questionnaire. Standard translation procedures were undertaken so that the survey is comparable between countries. Computer-assisted personal interview (CAPI) was used in half of the interviews in China, and the other half was done using paper and pencil. Mexico used only CAPI, while the other four countries used paper and pencil format for all interviews. The survey response rates ranged from 53% in Mexico to 93% in China. The population structure based on the United Nations Statistical Division was adjusted for with the use of sampling weights. The WHO Ethical Review Committee and local ethics research review boards provided ethical approval. All participants gave written informed consent.

### Mild cognitive impairment (MCI)

The recommendations of the National Institute on Aging-Alzheimer's Association were followed to identify people with MCI [16]. Identical algorithms utilized in previous SAGE publications were employed [17, 18]. In brief, fulfilling all following conditions corresponded to MCI:

(I) Impairment in at least one cognitive domain based on objective measures: corresponded to < -1 SD cut-off after adjustment for age, level of education, and country, on at least one of the following cognitive function tests: animal naming task [19], examining verbal fluency; word list immediate/delayed verbal recall of the Consortium to Establish a Registry for Alzheimer's Disease [19], assessing episodic and learning memory; digit span forward/backwards of the Weschler Adult Intelligence Scale [20], evaluating working and attention memory;
(II) Maintenance of functional ability independence: Questions on past 30-day basic activities of daily living (ADL) were used to assess this condition [21]: “How much difficulty did you have with eating (including cutting up your food)?” and “How much difficulty did you have in getting dressed?” Answering none, mild, or moderate to both of these questions referred to this condition, and all other people (i.e., those who answered ‘severe’ or ‘extreme’) were omitted from the analysis (935 participants aged 50 years or more).
(III) Worry regarding changing cognition: This was assessed by two questions: How would you best describe your memory at present?” and “Compared to 12 months ago, would you say your memory is now better, the same or worse than it was then?” Answering ‘bad’ or ‘very bad’ to the first question and/or responding ‘worse’ to the second corresponded to this condition.
(IV) Absence of dementia: People who were unable to participate in the survey due to severe cognitive impairment were omitted from the current study.

### Pain

“Overall in the last 30 days, how much of bodily aches or pain did you have?” was the question used to assess the level of pain with answer options ‘none’, ‘mild’, ‘moderate’, ‘severe’ and ‘extreme’. In the current analysis, ‘severe’ and ‘extreme’ were merged into one category as there were very few people who answered ‘extreme’. Furthermore, a dichotomized variable [i.e., severe/extreme pain (yes/no)] was also created and employed in some analyses.

### Mediators

The potential mediators examined in the current study included perceived stress, sleep/energy problems, and mobility limitations, and these were selected since they can be the consequence of pain, while they may also potentially increase future risk of cognitive decline [22–24]. Two questions each were used to assess these three conditions. The actual questions are described in supplementary Table S1. Each question was based on a five-point scale with answer options ranging from 'none' to ‘extreme/cannot do’ except for the two items on perceived stress, ranging from ‘never’ to ‘very often’. Based on the two questions for each individual condition, we utilized factor analysis with polychoric correlations to calculate a factor score, subsequently converted to scores ranging from 0 to 100, with higher values corresponding to worse health status [25].

### Control variables

Past literature was used as a guide to select the control variables [26], and these included age, sex, education (years), wealth quintiles based on income, marital status (married/cohabiting, never married, separated/divorced/widowed), past 30-day alcohol consumption, smoking (never, current, past), body mass index (BMI), number of chronic conditions, and depression. BMI was estimated as measured weight in kilograms divided by measured height in metres squared. BMI was categorized as underweight (BMI < 18.5 kg/m^2^), normal weight (BMI 18.5–24.9 kg/m^2^), overweight (BMI 25.0–29.9 kg/m^2^), and obesity (BMI ≥ 30 kg/m^2^) based on WHO guidelines [27]. Information on 10 chronic physical diseases (angina, stroke, arthritis, asthma, hearing problem, diabetes, edentulism, hypertension, visual impairment, chronic lung disease) were gathered. Table S2 (Appendix) provides complete details on how the diagnosis was determined. The number of chronic conditions was calculated per participant and categorized as 0, 1, and ≥ 2. Questions based on the World Mental Health Survey version of the Composite International Diagnostic Interview [28] were used for the endorsement of past 12-month DSM-IV depression.

### Statistical analysis

The statistical analysis was done with Stata 14.2 (Stata Corp LP, College station, Texas). The analysis was limited to people aged 50 years and over. The difference in sample characteristics was tested by Student’s* t*-tests and Chi-squared tests for continuous and categorical variables, respectively. Multivariable logistic regression analysis was performed to examine the association between severity of pain (four-category variable with values ‘none’, ‘mild’, ‘moderate’, ‘severe/extreme’) (exposure) and MCI (outcome). We also conducted test of trend to assess whether increasing severity of pain was dose-dependently associated with higher odds for MCI by including the variable on severity of pain as a continuous variable rather than a categorical variable in the model. The analysis was also stratified by age group (50–64 and 65 years and over) and sex. Next, to assess the degree of between-country heterogeneity in the association between severe/extreme pain and MCI, we conducted country-wise analysis and calculated the Higgin’s *I*^*2*^ which represents the level of heterogeneity that is not due to sampling error with a value of < 40% frequently viewed as negligible and 40–60% as moderate heterogeneity [29]. An overall estimate was calculated by meta-analysis with fixed effects based on country-wise estimates.

Finally, to quantify the degree to which perceived stress, sleep/energy problems, and mobility limitations may explain the association between extreme/severe pain and MCI, we conducted mediation analysis using the *khb* (Karlson Holm Breen) command in Stata [30]. This method can be used in logistic regression models and decomposes the total effect (i.e., unadjusted for the mediator) of a variable into direct (i.e., the effect of extreme/severe pain on MCI adjusted for the mediator) and indirect effects (i.e., the mediational effect). Using this method, the percentage of the main association explained by the mediator can also be calculated (mediated percentage). Each potential mediator was included in the model separately.

All regression analyses including the mediation analysis were adjusted for age, sex, education, wealth, marital status, alcohol consumption, smoking, BMI, chronic physical conditions, depression, and country, except for the sex- and country-stratified analyses which were not adjusted for sex and country, respectively. Adjustment for country was done by including dummy variables for each country in the model as in previous SAGE publications [17, 31]. The sample weighting and the complex study design were considered in all analyses. Results from the regression analyses are presented as odds ratios (ORs) with 95% confidence intervals (CIs). The level of statistical significance was set at *P* < 0.05.

## Results

**Fig. 1:**
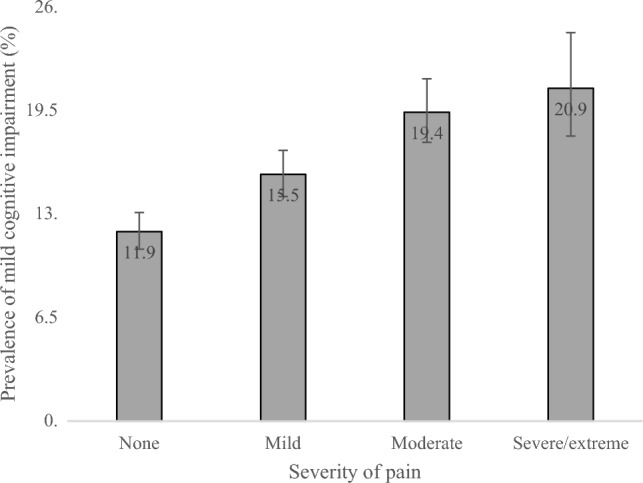
Prevalence of mild cognitive impairment by severity of pain. Bars denote 95% confidence intervals

**Fig. 2:**
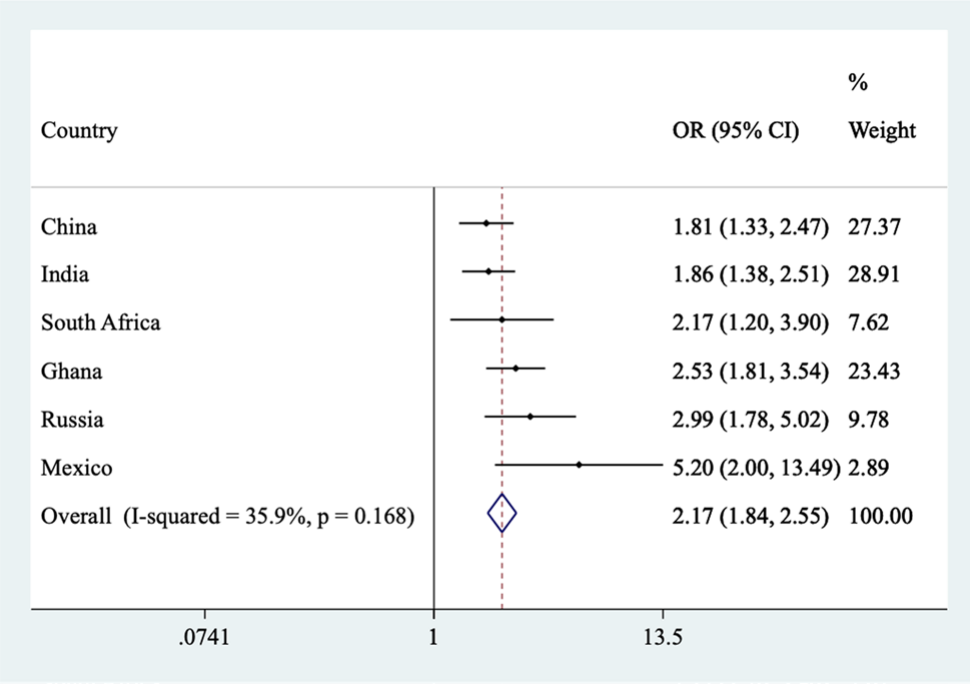
Country-wise association between severe/extreme pain and mild cognitive impairment (outcome) estimated by multivariable logistic regression. Abbreviation: *OR* odds ratio; *CI* confidence interval. Models are adjusted for age, sex, education, wealth, marital status, alcohol consumption, smoking, body mass index, chronic physical conditions, and depression. Overall estimate was obtained by meta-analysis with fixed effects

The final sample included 32,715 individuals (China *n* = 12,815; Ghana *n* = 4201; India *n* = 6191; Mexico *n* = 2070; Russia n = 3766; South Africa *n* = 3672) aged 50 years and over with preservation in functional abilities. The prevalence of MCI was 15.3%, while the prevalence of different severity of pain were: mild 32.7%; moderate 18.1%; severe/extreme 9.6%. The sample characteristics are shown in Table 1. The mean (SD) age was 62.1 (15.6) years and 51.7% were females. People with severe/extreme pain had much worse scores in terms of perceived stress, sleep/energy problems, and mobility limitations, compared to those without this condition. The prevalence of MCI increased linearly with increasing severity of pain (Fig. 1). For example, the prevalence of MCI was only 11.9% among those with no pain but this increased to 20.9% among those with severe/extreme pain. After adjustment for potential confounders, in the overall sample, compared to no pain, mild, moderate, and severe/extreme pain were dose-dependently associated with 1.36 (95% CI = 1.18–1.55), 2.15 (95% CI = 1.77–2.62), and 3.01 (95% CI = 2.36–3.85) times higher odds for MCI (Table 2). Age-stratified analysis showed that this association is slightly more pronounced among those aged 50–64 years, while sex-stratified analysis showed that the association is similar among males and females. Country-wise analysis showed that severe/extreme pain is significantly associated with higher odds for MCI in all countries with a negligible level of between-country heterogeneity (*I*^*2*^ = 35.9%) (Fig. 2). Finally, mediation analysis showed that perceived stress, sleep/energy problems, and mobility limitations explained 10.4%, 30.6%, and 51.5% of the association between severe/extreme pain and MCI (Table 3).

**Table 1:** Sample characteristics (overall and by severe/extreme pain)

Characteristic		Overall	Severe/extreme pain		*P*-value^a^
			No	Yes	
Age (years)	Mean (SD)	62.1 (15.6)	61.9 (15.5)	63.9 (15.7)	< 0.001
Sex	Female	51.7	50.5	62.8	< 0.001
	Male	48.3	49.5	37.2	
Education (years)	Mean (SD)	6.1 (8.9)	6.3 (8.9)	4.1 (7.8)	< 0.001
Wealth	Poorest	16.9	16.3	22.3	< 0.001
	Poorer	18.9	18.6	22.2	
	Middle	19.4	19.3	20.2	
	Richer	21.5	21.8	18.3	
	Richest	23.3	24.0	16.9	
Marital status	Married/cohabiting	76.3	77.4	66.3	< 0.001
	Never married	1.7	1.8	1.5	
	Separated/divorced/widowed	22.0	20.9	32.1	
Alcohol consumption	No	80.9	80.1	88.4	< 0.001
	Yes	19.1	19.9	11.6	
Smoking	Never	58.7	59.2	53.8	0.008
	Current	34.9	34.4	39.9	
	Past	6.4	6.4	6.3	
Body mass index	Underweight	16.2	15.0	28.1	< 0.001
	Normal	47.8	48.4	41.9	
	Overweight	24.5	25.1	17.8	
	Obese	11.5	11.5	12.2	
No. of chronic physical	0	26.5	27.8	15.3	< 0.001
conditions	1	36.4	37.4	27.3	
	≥ 2	37.0	34.9	57.3	
Depression	No	94.5	96.1	79.7	< 0.001
	Yes	5.5	3.9	20.3	
Perceived stress^b^	Mean (SD)	39.6 (40.3)	38.4 (39.8)	50.9 (39.8)	< 0.001
Sleep/energy problems^b^	Mean (SD)	26.5 (44.1)	23.9 (42.0)	50.8 (42.0)	< 0.001
Mobility limitations^b^	Mean (SD)	31.4 (45.1)	28.1 (42.2)	61.9 (36.8)	< 0.001

Abbreviation: *SD* standard deviation

^a^*P*-value obtained by Chi-squared tests and Student’s *t*-tests for categorical and continuous variables, respectively

^b^Scores ranged from 0 to 100 with higher scores representing worse health status

**Table 2:** Association between pain and mild cognitive impairment estimated by multivariable logistic regression (overall and by age groups or sex)

Pain	Overall		Age (years)				Sex			
			50–64		≥ 65		Male		Female	
	OR	95% CI	OR	95% CI	OR	95% CI	OR	95% CI	OR	95% CI
None	1.00		1.00		1.00		1.00		1.00	
Mild	1.36***	[1.18, 1.55]	1.50***	[1.28, 1.77]	1.14	[0.90,1.44]	1.24*	[1.02, 1.52]	1.46***	[1.22, 1.76]
Moderate	2.15***	[1.77, 2.62]	2.47***	[2.02, 3.04]	1.72**	[1.24,2.37]	2.13***	[1.52, 2.99]	2.18***	[1.77, 2.69]
Severe/extreme	3.01***	[2.36, 3.85]	3.08***	[2.19, 4.34]	2.57***	[1.78,3.71]	3.05***	[2.04, 4.55]	3.01***	[2.19, 4.14]

Abbreviation: *OR* odds ratio; *CI* confidence interval

Models are adjusted for age, sex, education, wealth, marital status, alcohol consumption, smoking, body mass index, chronic physical conditions, depression, and country, with the exception of the sex-stratified analysis which was not adjusted for sex

Significant test for trend for all models (*P* < 0.001)

*p < 0.05, **p < 0.01, ***p < 0.001

**Table 3:** Mediators in the association between extreme/severe pain and mild cognitive impairment

Mediator	Effect	OR [95% CI]	*P*-value	%Mediated
Perceived stress	Total	2.03 [1.63, 2.53]	< 0.001	10.4
	Direct	1.89 [1.52, 2.34]	< 0.001	
	Indirect	1.08 [1.04, 1.11]	< 0.001	
Sleep/energy problems	Total	2.02 [1.62, 2.52]	< 0.001	30.6
	Direct	1.63 [1.30, 2.04]	< 0.001	
	Indirect	1.24 [1.18, 1.31]	< 0.001	
Mobility limitations	Total	2.07 [1.66, 2.58]	< 0.001	51.5
	Direct	1.42 [1.13, 1.79]	0.003	
	Indirect	1.45 [1.35, 1.57]	< 0.001	

Abbreviation: *OR* odds ratio; *CI* confidence interval

Models are adjusted for age, sex, education, wealth, marital status, alcohol consumption, smoking, body mass index, chronic physical conditions, depression, and country

## Discussion

### Main findings

Increasing severity of pain was dose-dependently associated with higher odds for MCI. For example, in the overall sample, compared to no pain, severe/extreme pain was significantly associated with 3.01 times higher odds for MCI. Severe/extreme pain was significantly associated with higher odds for MCI in all countries included in the study, with between-country heterogeneity being of a negligible level. Mobility limitations explained more than half of the association between pain and MCI, followed by sleep/energy problems (30.6%), and perceived stress (10.4%). To the best of our knowledge, this is the first study on the association between pain and MCI in LMICs as well as the first to identify its potential mediators.

### Interpretation of the findings

There are several mechanisms that may explain the link between pain and MCI. First, mobility limitations explained more than half of the association between pain and MCI. Pain may lead to mobility limitations via, for example, avoidance of activities of daily living and physical activity that may exacerbate pain [7]. Thus, mobility limitations may lead to low levels of physical activity, and this is a known risk factor for cognitive decline [32]. Apart from this, mobility limitations (e.g., gait dysfunction) per se may increase risk for MCI via the presence of neurofibrillary tangles in the substantia nigra as well as leading to decreased cognitive stimulation more generally [8]. Second, sleep/energy problems explained more than 30% of the association between pain and MCI. Pain may induce sleep problems via an increase in depressive symptoms [33] as well as difficulty falling asleep and movement during sleep [5]. In turn, sleep problems may lead to higher risk of MCI through the accumulation and impaired clearance of toxic metabolites in the brain [6] or inflammation [34, 35]. Next, perceived stress was also identified as a potential mediator but to a lesser extent. Pain can lead to perceived stress not only via pain itself but also through its impact on quality of life [4]. Moreover, pain and stress share significant conceptual and physiological overlaps both challenging the body’s homeostasis [4]. Perceived stress has been hypothesized to increase risk for MCI through mechanisms such as dysregulation of hormones (e.g., cortisol) and increased production of pro-inflammatory cytokines, which can impair the neural structure and function implicated in cognitive performance [36]. Finally, apart from these pathways, pain may affect brain plasticity and cause structural changes in different cortical regions associated with cognition [12].

### Implications of the study findings

The present study suggests that pain may be a modifiable risk factor for cognitive impairment among middle-aged and older adults in LMICs, and that addressing pain may prevent cognitive impairment in this context. Directly mitigating pain may potentially have a large impact especially in the context of LMICs as more than 80% of the global population lacks access to treatments for moderate to severe pain, and the majority of these individuals live in LMICs [3]. Indeed, organizations such as the International Association for the Study of Pain and the World Federation of Societies of Anesthesiologists are working with national professional societies to develop local expertise and leadership for pain management. Furthermore, it will be important for research to expand also in services including non-pharmacological based therapies such as music therapy [37], especially for areas where access to health facilities or analgesics is limited. Finally, considering the role of mobility, workers delivering pain treatments should be able to promote and to support people to address barriers to physical activity.

### Strengths and limitations

The use of a large nationally representative dataset from six LMICs and the identification of potential mediators in the pain/MCI relationship are clear strengths of the present study. However, findings must be interpreted in light of their limitations. First, the study is cross-sectional in nature, and thus, causality or temporal associations cannot be determined. In relation to this, it is possible for the mediational effect calculated in our study to be an overestimation due to the various pathways that may exist between pain, MCI, and the potential mediators. Second, we had no information on analgesic drugs. Opioids or anticholinergic antidepressants such as amitriptyline may themselves lead to cognitive decline [38], and thus, we were unable to know the extent to which the association between pain and cognitive decline may be explained by use of analgesics. Finally, our study was not designed to clinically diagnose dementia. Therefore, while it is possible for some people with mild dementia to have been included in our sample, the prevalence of MCI in our study was within previously reported figures [39].

## Conclusion

In this large representative sample of middle-aged to older adults from six LMICs, pain was associated with MCI dose-dependently. Moreover, mobility limitations and sleep/energy problems accounted for a large proportion of the association. Addressing pain among middle-aged and older adults may aid in the prevention of MCI and ultimately dementia in the context of LMICs, but future longitudinal and intervention studies would be necessary to confirm this.

## Data Availability

The data that support the findings of this study are available from the corresponding author upon reasonable request.
